# Characterizing Critical Sources of Carbon Emissions Using Principal Component Analysis

**DOI:** 10.1155/tswj/6175776

**Published:** 2026-04-13

**Authors:** Moiz Qureshi, Muhammad Ismail, Muhammad Daniyal, Inzamamul Haq, Kassim Tawiah, Francis Kwame Bukari, Richard Kwame Ansah, Killian Asampana Asosega

**Affiliations:** ^1^ Department of Statistics, Quaid-i-Azam University, Islamabad, 45320, Pakistan, qau.edu.pk; ^2^ Department of Statistics, University of Sindh, Hyderabad, 76080, Pakistan, usindh.edu.pk; ^3^ College of Statistical Sciences, University of the Punjab, Lahore, Pakistan, pu.edu.pk; ^4^ Department of Statistics, The Islamia University of Bahawalpur, Bahawalpur, Pakistan, iub.edu.pk; ^5^ Department of Statistics, Abdul Wali Khan University, Mardan, Pakistan, awkum.edu.pk; ^6^ Department of Mathematics, Saveetha School of Engineering (SIMATS), Thandalam, 600124, Chennai, Tamil Nadu, India, saveethaengineering.com; ^7^ Department of Mathematics and Statistics, University of Energy and Natural Resources, Sunyani, Ghana, uenr.edu.gh; ^8^ Department of Statistics and Actuarial Science, Kwame Nkrumah University of Science and Technology, Kumasi, Ghana, knust.edu.gh

**Keywords:** carbon dioxide (CO_2_) emissions, environmental management, policy formulation, principal component analysis, sustainable development

## Abstract

The emerging issue of carbon dioxide (CO_2_) emissions is highly affecting global sustainable and economic development endeavors. Countries with high population growth, rapid industrialization, and significant energy needs find themselves in this bracket. This study, based on data from 1960 to 2018, evaluates carbon emissions using principal component analysis (PCA). The findings indicate that two leading principal components (C.1 and C.2) had the greatest impact as they accounted for seventy‐seven percent (77%) of the total variance. The eigenvalues of both components were greater than one, signifying their significance. C.1 shows a strong connection for CO_2_ emissions, total population, and production of electric energy through various sources. C.2 is more connected to the growth of industries. The scree plot confirms this by finding them to be dominant. This emphasizes the interaction between electricity production, specifically from coal, and the demographic data. The results highlight how PCA can be utilized to distinguish drivers that cause the emission of carbon to provide an understanding that might be used in managing the environment and setting relevant policies.

## 1. Introduction

The consequences of increased carbon dioxide (CO_2_) emissions are complex. The environmental effects of excessive CO_2_ emissions include worsening climate, which results in new climatic changes, an increase in temperatures, and other negative ecological effects [1]. Such changes may interfere with agricultural activities, affect rainfall patterns, flow of rivers, and destabilize the overall environmental balance. Unregulated CO_2_ emissions may have dire consequences on a country’s economic fortune. This includes low agricultural production, elevated healthcare expenses occasioned by health complications resulting from air pollution, and the susceptibility of infrastructures to climatic hazards [2]. Additionally, with increasing global consciousness of sustainability and environmental responsibility, the inability to mitigate the issue of CO_2_ emissions could cause poor global reputation, trade‐related restrictions, and limited access to the global market of goods and services [3]. The National Petroleum Council of the United States has predicted a massive increment in the global energy demand, assuming that the rate of growth will be fifty to sixty percent by 2030 [4].

Yuan et al. [5] made good contributions to the issue of the complex dynamics of urban and rural households’ carbon emissions in China. Their analyses enlightened policies and general strategies that can be used to reduce the amount of carbon emissions, promote sustainable development, and reduce the environmental effect of household actions and consumption behavior [5]. Li and Wang [6] discovered that economic factors of urbanization, gross domestic product (GDP) per capita, industry structure, and technology topped the list of factors that influence CO_2_ emissions. They highlighted the importance of integrating these factors into consideration when designing strategies to reduce emissions and ensure sustainable development, either locally or even internationally [6].

The gray model was used by Pao and Tsai [7] to model CO_2_ emissions in Brazil between the years 1980 and 2007. They built and implemented a combination of models, which constitute optimization algorithms such as quantum harmony and discounted mean square, to forecast CO_2_ emissions in the top five countries producing maximum CO_2_ emissions on the planet [8]. Auffhammer and Carson [9] came up with a roadmap of the CO_2_ emissions of China by examining the provincial panel information [10–13]. They used the standard gray model and an improved version in predicting CO_2_ emissions [14, 15].

There have been many studies on how several variables are interdependent in the theory of CO_2_ emissions, both qualitatively and quantitatively [16]. Al‐Mulali and Normee Che Sab [17] also explored the effect of simple energy use on CO_2_ emissions in 16 developing countries, particularly attempting to determine its influence on the economic growth of those countries. Wang et al. [18] examined critical aspects contributing to the production of CO_2_ in the road freight transport sector in China. Zhang and Nian [19] estimated the CO_2_ emission rates and examined the causes of the same in China’s transportation sector. Lin et al. [20] presented a new bootstrap autoregressive distributed lag (ARDL) bounding test used to determine the relationship between development and CO_2_ emissions.

On the other hand, Pao and Tsai [21] investigated the relationship between cause and effect involving the number of pollutants released, energy consumption, and economic production in different countries over a given period. The results in India indicate that there is a one‐way causal relationship between economic growth and CO_2_ emissions within the short term, whereby economic growth increases the level of CO_2_ emissions. Also, energy consumption has a causality relationship with economic growth that is one‐way [22]. Results from Russia suggest that both economic growth and energy conservation strategies can mitigate emissions without adversely affecting economic development. The causality results showed that there is a bidirectional strong Granger causality between output, the consumption of energy, and emissions, and when an abrupt change occurs in the framework, each variable performs a short‐run adjustment to re‐establish the equilibrium over the long run [23]. Empirical data for China between 1960 and 2007 showed unidirectional Granger causality from GDP to energy consumption, and from energy consumption to carbon emissions in the long term. Evidence suggests that neither carbon emissions nor energy use drives economic growth [24].

In the eventual over time, within Brazil, Russia, India, and China (BRIC), CO_2_ emissions tend to be dynamic in terms of energy consumption and inelastic in terms of foreign direct investment (FDI), supporting the Environmental Kuznets Curve (EKC). The causality results show that there is strong bidirectional causation between emissions and FDI, as well as strong translational dependency from output to FDI. The evidence appears to corroborate the pollution haven hypothesis, as well as the halo and scale impacts [25].

Within BRICS (Brazil, Russia, India, China, and South Africa), with regard to the electricity–GDP relationship, there is evidence to support the feedback hypothesis for Russia and the conservation hypothesis for South Africa from the empirical evidence [26]. Nevertheless, Brazil, India, and China have a neutrality hypothesis, which indicates that neither electricity consumption nor economic growth reacts to the other in these three countries. With regard to the GDP–CO_2_ emissions relationship, a feedback hypothesis for Russia, unidirectional Granger causality from GDP to CO_2_ emissions in South Africa, and a reverse relationship of CO_2_ emissions to GDP in Brazil have been established. No evidence of Granger causality is found in the relationship between GDP and CO_2_ emissions in India and China. Furthermore, electricity consumption Granger causes the emissions of CO_2_ in India, but no Granger causality from the consumption of electricity to the emission of CO_2_ exists in Brazil, Russia, China, and South Africa. Therefore, the different findings for the BRICS countries imply that policies cannot be used uniformly because they will have different effects in each of the BRICS countries studied [26].

Studies in the Middle East countries contradicted the EKC hypothesis [27]. Ozcan [27] discovered evidence in favor of the U‐shaped EKC in five Middle Eastern countries, but only three showed an inverted U‐shaped curve. In addition, income and emissions do not appear to be linked in the remaining four countries. In the short run, there appears to be a unidirectional causality from economic growth to energy consumption. Notwithstanding, in the long run, the unidirectional causality chain extends from energy consumption to emissions [27].

Studies from the OECD countries (Organization for Economic Co‐operation and Development) showed that environmental taxes harm carbon emissions, whereas economic expansion impedes environmental quality by increasing carbon emissions. Furthermore, renewable energy consumption, environmental technologies, and financial development enhance environmental quality by lowering carbon emissions [28].

Umer et al. [29] discussed greenhouse gas (GHG) emissions in the electricity industry in Pakistan, focusing on CO_2_, CH_4_, and N_2_O rather than the popular CO_2_. Iftikhar et al. [30] outlined a detailed analysis and prediction of emissions of CO_2_ in Pakistan through different combinations of hybrid approaches in regression and time series. Using models of novel gray relational analysis (GRA), Rehman et al. [31] examined how high CO_2_‐emitting sectors in Pakistan are influenced by energy consumption, economic development, and population growth (PGR).

It is worth mentioning that several earlier studies channeled efforts in modeling and predicting a single component of decomposition [32]. The disadvantage, nonetheless, is that such an approach increases the complexity of the computations, and this could result in lower modeling efficiency. Issues relating to the computational complexity and the modeling efficiency highlight the importance of a simplified and efficient forecasting procedure [33]. There should be a balanced solution to ensure that there are correct predictions to reduce the pressures of complex modeling processes. Ensuring efficiency to the maximum is important in order to make informed decisions in the ever‐changing world of carbon emissions and pricing [34]. Proper estimation and measurement of GHG emissions would be key to a low‐carbon future, particularly in the power sector and other emission hotspots [35, 36]. Investigation into the integration of renewable energy systems and reduction of carbon emissions linked with transportation focuses on the shift to a clean and, therefore, more sustainable energy environment [29, 37]. Forecasting has gained more significance in sustainability and policy planning of clean energy sources [38, 38]. An aspect that has become more important in economic and environmental applications over the last few years [39–41]. While current carbon emission forecasting models and studies have demonstrated commendable predictive capabilities, there remains room for enhancement in certain aspects.

Researchers and professionals in various fields have focused their efforts on complex studies of energy usage trends, the identification of cleaner and renewable energy sources, improvements in energy efficiency, and the development of new technologies that reduce carbon emissions [42–44]. Most of these efforts have concentrated on renewable energy and related policy implications [29, 45, 46]. This study aims to strike a balance between energy and environmental sustainability demands.

Recent studies have further emphasized the critical role of technological innovation and sector‐specific strategies in decarbonization. For instance, research on the optimal design of renewable‐driven polygeneration systems highlights novel approaches for integrating sustainable energy solutions, which are directly relevant to transitioning national energy mixes [46]. Furthermore, analyses of modern home design and load management systems demonstrate how demand‐side efficiency and residential energy consumption patterns significantly impact national emissions profiles, offering actionable insights for policy [45]. In the context of industralized, non‐indutralized or populated countries, detailed assessments of GHG emissions in the power sector provide a crucial roadmap and benchmark for understanding sectoral contributions and mitigation potentials, underscoring the importance of accurate emissions estimation for effective policy formulation [29]. These evolving research streams reinforce the need for robust analytical frameworks, such as the principal component analysis (PCA) employed in this study, to distil complex, multidimensional emission drivers into actionable intelligence for sustainable development.

This study summarizes and prioritizes the most effective elements derived from the data on carbon emissions. Selective attention to the factors that most strongly influence changes in carbon emissions can help provide more efficient modeling without sacrificing accuracy. PCA permits most of the original variance to be characterized as the numerous features are reduced tremendously by transforming the information into a new coordinate frame based on the key components. The process of modeling and calculations becomes simpler and more efficient.

The next sections provide the source of the data and variables included, methods, results, and their discussion, and the conclusion with policy recommendations based on the results.

## 2. Data and Methods

### 2.1. Data

The dataset utilized in this study comprises various variables for Pakistan from 1960 to 2018. These variables include total population (TP), population growth rate (PGR) (annual %), electricity production from coal sources (EPCS) (% of total), GDP (annual growth), CO_2_ emissions (CEs) (metric tons per capita), and electricity production from oil, gas, and other sources (EPOGS) (% of total), and industry (including construction) value added (IVG) (% of GDP). The data for this study were obtained from the World Bank’s data indicators at https://databank.worldbank.org/country/PAK/556d8fa6/Popular_countries#selectedDimension_WDI_Ctry.

### 2.2. Methods

#### 2.2.1. Preprocessing and Standardization

To improve the methodological transparency and reproducibility, a detailed description of the data preprocessing stage is provided. In particular, the standardization of all variables was performed with the z‐score normalization method to provide comparability of indicators measured on dissimilar scales. This transformation normalizes each variable to a mean of zero and a standard deviation of one. This avoids the use of variables with large component bias in the extraction of the principal components. The formulation of standardization is given by

(1) z=x−x¯sx,

where *z* denotes the standardized values, *x* denotes the values of each variable, $\overset{¯}{x}$ denotes the sample mean, and *s*_*x*_, the sample standard deviation. This shows that the results of the PCA are based on a balanced dataset where all indicators contribute equally to the formation of components.

#### 2.2.2. PCA

Dimensionality reduction is the process of converting high‐dimensional data into a meaningful representation with decreased dimensionality [32]. PCA is one of the dimensionality reduction techniques that extracts fewer principal components (C.) as a linear combination of the original variables. The primary objective of PCA is to get significant information from the data and express it as a set of new orthogonal variables known as C.s. Every C. is orthogonal to the others and is a linear combination of the initial variables (with some remaining correlation). The highest proportion of data variability is explained by the first C., which is a mathematical combination of measurements. Additionally, C.s iteratively express much of the overall variance in the data as they can, explaining more of the variation in the data, and so on. This explains why few describe the variation of a large number of original responses.

Z‐score normalization was applied to all continuous variables before PCA to ensure comparability across different measurement scales. Because PCA is sensitive to variable magnitude and the dataset comprises features expressed in diverse units, standardization prevents any single variable from disproportionately influencing the principal components and ensures equal contribution from all variables [33, 34, 47]. Geographical statistical analysis was subsequently employed to evaluate spatial patterns, provide local contextual insight, and validate the robustness of the PCA results. Subsequently, positive matrix factorization (PMF) was applied to further characterize pollution source types and quantify their relative contribution rates, building upon the findings of the preceding analyses. The combined application of PCA, geographical statistical analysis, and PMF proved highly effective for pollution source apportionment, offering a comprehensive understanding of pollution origins and their associated impacts [48].

Mathematically, for a dataset with *p* variables, *X*_1_, *X*_2_, …, *X*_*p*_, the *P* *C*_*i*_ is

(2) PC1=w11X1+w12X2+⋯+w1pXp.

## 3. Results and Discussion

The descriptive statistics for each variable are shown in Table 1. These included the mean, median, maximum, and lowest values of the data as well as the standard deviation (Std. Dev.), skewness, and kurtosis of the data.

**TABLE 1:** Descriptive statistics of the seven study variables.

Study variable	Minimum	Maximum	Mean	Median	Std. Dev.	Skewness	Kurtosis
CEs	0.31	0.98	0.57	0.57	0.19	0.27	−1.15
PGR	2.06	3.36	2.67	2.69	0.39	0.11	−1.02
TP	44.99	212.23	112.69	104.51	51.39	0.38	−1.15
GDP	0.47	11.35	5.22	5.07	2.31	0.26	0.30
IVG	14.57	22.93	20.01	20.15	1.79	−0.70	0.42
EPCS	0.02	1.22	0.49	0.24	0.45	0.72	−1.17
EPOGS	38.00	71.83	55.19	50.87	9.44	0.04	−1.30

**FIGURE 1:**
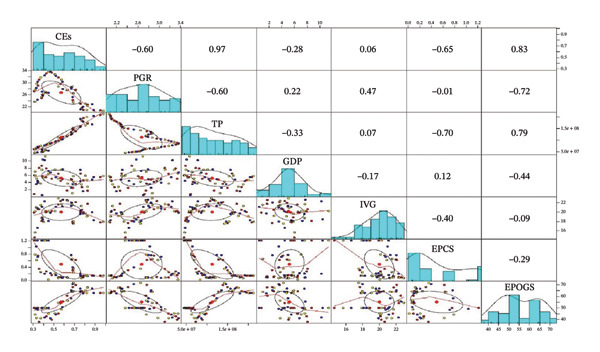
Pearson’s correlation of the study variables.

In Figure 1, significant statistical relationships were observed among various variables related to CO_2_ emissions . Notably, a positive correlation (*r* = 0.97) was observed between CEs and the TP. This indicates that as the population increases, there is a substantial concurrent rise in CO_2_ emissions. In essence, a larger population size corresponds to higher CO_2_ emissions. Moreover, a weak positive correlation emerged between IVG and CEs. This suggests that the expansion of industrial sector has a limited impact on CO_2_ emissions, indicating a minor influence of industrial activity on emissions. Conversely, a moderate negative correlation was identified between electricity EPCS and CEs, implying that as CEs increase, there is a gradual reduction in electricity generation from coal sources. The observed negative correlation between EPCS and per‐capita emissions in the dominant component may seem contradictory given coal’s carbon intensity. However, this can be interpreted in the context of energy history. The initial rapid growth in per‐capita emissions (1960s–1990s) was likely fueled by increased use of oil and gas for transport, industry, and power. The more recent rise in coal’s share for power generation (post–2000s) may have coincided with slower per‐capita emission growth rates due to efficiency improvements, fuel switching in other sectors, or the deployment of newer coal plant technology relative to older oil/gas infrastructure.

Clusters of closely connected variables are highlighted in the correlation matrix, and these groupings correspond exactly with the main component loading clusters. The underlying structure that PCA captures is shaped by the tendency for variables with strong positive or negative correlations to load together. From correlations to component generation, this process makes the environmental dimensions that each component represents more understandable. By analyzing these clustered variables, one can transform statistical trends into insights that are pertinent to policy, like identifying important environmental pressures, monitoring priorities, and possible intervention targets. This nuance highlights a key strength of PCA: it reveals statistical associations in the data that merit qualitative, context‐driven explanation, moving beyond simplistic assumptions. Figure 1 reveals a trade‐off where higher CEs are linked to decreased coal‐based electricity production.

The existence of a correlation between the study variables warrants the application of PCA for greater variability in identifying more relevant and fewer variables. The procedure computes eigenvalues as well as eigenvectors. As shown in Table 2, the first two eigenvalues are greater than one. This means that the first two principal components are retained because they explain greater variability in the data. This indicates that the first two principal components are enough to explain all the study variables.

**TABLE 2:** Eigenvalues of the principal components.

C.1	C.2	C.3	C.4	C.5	C.6	C.7
3.71	1.68	0.95	0.38	0.18	0.07	0.02

The use of eigenvectors in PCA is crucial as it provides a clue to the direction and contribution of each of the principal components in the original feature space. Essentially, they can be explained as coefficients or weights given to every variable in the dataset. In interpreting these eigenvectors, the signs as well as the magnitudes result in useful information about the contribution made by each variable in the respective principal component. To connect each primary component to actual emission drivers, the eigenvectors were interpreted. Shifts toward cleaner energy can reduce emission intensity, as demonstrated by components dominated by energy‐associated variables. Similarly, elements with significant contributions from industrial value‐added factors show how changes in efficiency and production structure affect mitigation approaches. It is easier to understand how structural and sectoral modifications promote actual environmental policy actions when these loadings are linked to realistic emission causes.

Additionally, these results are interpreted in detail, as about the first principal component (C.1), which is one of the data points that were obtained from the dataset. It is interesting to note that PGR, GDP, and EPCS indicate negative values in the eigenvector elements. These negative signs indicate an inverse relationship between these variables and C.1. On the other hand, CEs, TP, and EPOGS share the same‐sized eigenvector elements with positive values of the first principal component. It means that these variables have a positive relationship with C.1. Interestingly, IVG does not contribute to the variation that is explained by C.1, because the eigenvector weight has a value of zero as indicated in Table 3. This wisdom of the strength of the variable determinants can be useful in explaining the covert configuration of the information and can inform the forthcoming examination or decision‐making actions. In particular, the C.1, which accounts for the highest percentage of variance, was observed to have high positive loadings on carbon emissions, TP, and energy consumption, which indicates the population‐based and demand‐based side of the emissions. The second principal component (C.2) had high loadings of industrial value added and the fuel mix, meaning that industrial activity and fuel composition are crucial determinant factors of variability of the emissions. These statistical clues underscore the fact underpinning the point for the consideration of actions like enhancing renewable energy adoption, better energy efficiency measures, supporting sustainable industrial operations, and the shift to cleaner production processes to mitigate the primary sources of emissions.

**TABLE 3:** Eigenvalues of the variables under study.

Variables	C.1	C.2	C.3	C.4	C.5	C.6	C.7
CEs	0.5	−0.1	0.2	−0.1	0.2	0.6	0.6
PGR	−0.4	−0.5	0.0	0.2	0.7	0.3	−0.2
TP	0.5	−0.1	0.1	0.1	−0.1	0.3	−0.8
GDP	−0.2	0.1	0.9	−0.4	0.1	−0.1	−0.1
IVG	0.0	−0.7	−0.2	−0.6	−0.3	0.0	0.0
EPCS	−0.3	0.5	−0.3	−0.5	0.1	0.5	−0.2
EPOGS	0.5	0.2	−0.1	−0.4	0.6	−0.5	−0.1

**FIGURE 2:**
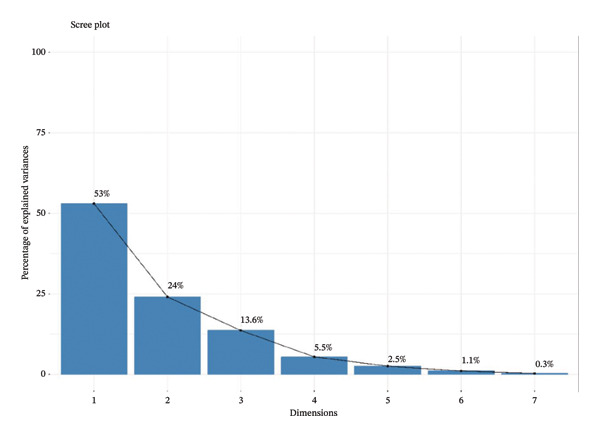
Scree plot.

In Figure 2, the plot declines at the beginning with a sharp fall in eigenvalues or variance accounted for, after which the decline is gradual. The components or factors to keep are normally identified by the location of the “elbow” point on the plot where the very steep drop‐off becomes level. The rest of the eigenvalue or variance explained by the remaining variables is very small. This fact is a justifiable compensation given that the information contained in the data is likely to be captured in just a few dimensions. Plot‐wise, the observation reveals the representatives of the component numbers on the vertical axis, and the percentage inequality is clarified on the horizontal axis. The first observation is that the values are generally declining at a very rapid rate, meaning that the initial components or factors represented the largest proportions of the variance in the data. It is the sharp decline that is caused by the most powerful trends or frameworks within the data set. The variance explained by the horizontal axis decreases as the curve moves further along the horizontal axis. This is because later components or factors explain a smaller percentage of the variance, and hence, are less significant and less important in the data. Such components are termed noise. Since the elbow point cross‐cuts its direction on the second major component, it leaves two factors as a trade‐off between attaining maximum information of importance in the data and unnecessary complexity.

Table 4 shows the proportion of variance explained by each C. in the data. This is a critical metric that quantifies the extent to which each C. captures information within the dataset relative to the total variability present. In this context, C.1, with a proportion of 0.53, signifies that it explains a substantial 53% of the total variability in the data. Similarly, C.2, with a proportion of 0.24, accounts for 24% of the variation in the data. Conversely, C.7, with a proportion of 0.003, represents a mere 0.3% of the dataset’s variation, indicating its limited importance in summarizing the data. Moreover, the cumulative proportion of explained variance is invaluable in determining how many principal components to retain when conducting dimensionality reduction. In this case, the cumulative proportions of C.1 and C.2 amount to 77%. This suggests that by retaining these two components, a significant portion of the dataset’s variability (77%) can be preserved while achieving the desired level of data compression or dimensionality reduction. This strategic selection of principal components not only simplifies the dataset but also retains the most informative aspects, ensuring that the essential patterns and relationships within the data are still adequately represented.

**TABLE 4:** Importance of the components by SD, PV, and CP.

Importance of components	C.1	C.2	C.3	C.4	C.5	C.6	C.7
Standard deviation (SD)	1.926	1.297	0.976	0.618	0.422	0.273	0.143
Proportion of variance (PV)	0.530	0.240	0.136	0.055	0.025	0.011	0.003
Cumulative proportion (CP)	0.530	0.770	0.906	0.961	0.986	0.997	1.000

**FIGURE 3:**
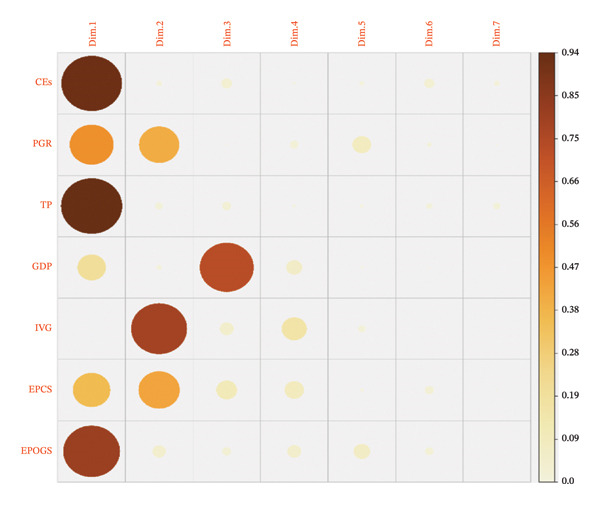
Loading plot capturing the variation of each variable.

The loading plot in Figure 3 is an important tariff used in clarifying the correlation among C.s and original variables in the multivariate data. The plot indicates that C.1 has strong relationships with three important original variables, CEs, TP, and EPOGS. This means that these variables may have a common setup in that, as C.1 rises, these variables also demonstrate coincident workings. Also, about 50% of the variance in PGR and EPCS is captured in C.1. As a result, differences in C.1 are related to specific degrees with the changes in PGR and EPCS. On the other hand, C.2 largely coincides with changes in IVG and in the same way, accounts for approximately half the variation that exists between PGR and EPCS. Therefore, the changes in C.2, in some way, reflect in PGR and EPCS. Lastly, C.3 is uniquely defined by the fact that it absorbs the entire inconsistency of the variable GDP. This indicates that C.3 variations have a direct and fast attachment to changes in GDP and remain comparatively slight with the rest of the variables.

**FIGURE 4:**
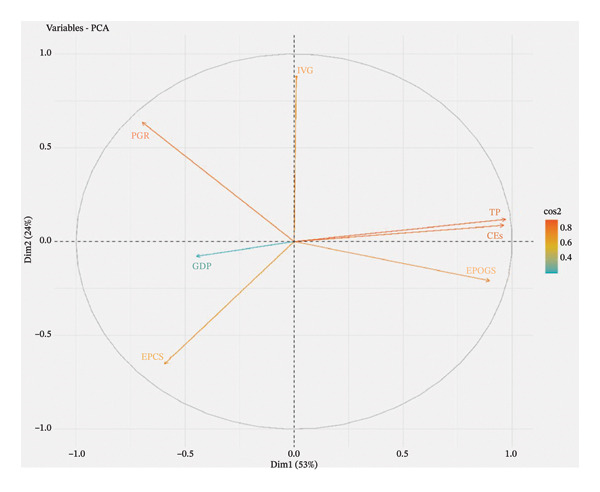
Biplot of the seven study variables by C.1 and C.2.

In Figure 4, it is worth noting that CEs, TP, and EPOGS fall near the circle in the positive direction of C.1. This position implies that these variables are highly driven and delimited by C.1. They move in the direction of C.1. In the same manner, IVG is represented by a long vector toward the positive direction of C.2. This shows that it contributes significantly to C.2. Conversely, EPCS lies between C.1 and C.2, implying that both components contribute to its variability. Moreover, the GDP variable is skewed toward the negative a little bit less than C.2. This placement signifies the fact that C.2 does affect GDP, but to a rather small degree, as it does not follow the path of this main element. The correlation analysis showed that there is a close positive relationship between carbon emissions, PGR, and total consumption of energy. This is reflected in the highest loadings in the first principal component. This means that the former component is mostly a reflection of the joint effect of the demographic growth and the energy demand on the emission patterns. Similarly, the second major component that has high loadings on the indicators of industrial value added and energy mix captures the contribution of industrial activities in the variability of emissions. Such statistical interrelations indicate that strategies to reduce emissions can be directed to demand‐side energy efficiency, clean energy transitions, and industrial modernization.

### 3.1. Comparative Insight

This study compared PCA‐based emission patterns with findings from research on other developing economies, specifically those in the BRICS, Association of Southeast Asian Nations (ASEAN), and Middle East and North Africa (MENA) regions, to place our findings in a broader context [49–51]. Similar to these studies, the main components of this study emphasize how industrial activity, economic expansion, and energy structure have a major impact on emission trends. Nonetheless, country‐specific factors, including varying energy transitions, industry makeup, and policy responsiveness, are reflected in the proportional weight of these drivers. This comparative viewpoint supports the applicability of our findings and harmonizes them with well‐established data from similar economies [52, 53].

Additionally, the scree plot and the first two PCs (PC1 = 53% and PC2 = 24%) are the most significant for interpretation because they account for the majority of the total variation. This is further supported by the biplot, which shows how C.1 is dominated by fossil‐energy and emission‐intensive variables, such as petroleum consumption, CO_2_ emissions from solid fuels, and electricity generation from coal, gas, and oil, demonstrating heavy reliance on fossil‐based power. C.2, on the other hand, records structural and demographic factors, such as sectoral power consumption, population increase, and industrial value added. The scree plot and biplot together demonstrate that, in addition to addressing population‐driven demand pressures, mitigation programs should prioritize switching from fossil fuel‐based electricity to renewable sources and enhancing industrial energy efficiency [54]. Sustainable Development Goals (SDGs) 7 and 13 demand population‐driven planning, energy efficiency, and the expansion of renewable energy sources,which is evident in the first component (population, energy usage, and CO_2_). Cleaner fuels, electrification, energy audits, and industrial decarbonization are captured in the second component (industrial structure, energy mix) [55, 56], thereby aligning with SDGs 9 and 13.

## 4. Conclusion

This study involved a thorough examination of the major variables that contribute to carbon emissions from 1960 to 2018, utilizing data from Pakistan as a case study. The choice of these factors of influence was an elaborate procedure, covering CEs per capita, TP, annual GDP growth, PGR rate, value added by the industry (including construction) as a percentage of GDP (IVG), share of electricity EPCS in total, and share of electricity production from oil, gas, and other sources (EPOGS) in the total energy mix. The attributes of these were analyzed through principal component analysis (PCA). Results show that two principal components, principal component 1 (C.1) and principal component 2 (C.2), were instrumental in accounting for most of the variance in the data, each contributing to fifty‐three percent and twenty‐four percent, respectively. The eigenvalues of C.1 and C.2 were higher than the threshold value of one, which means that the two components play an important role in the explanation of the original data. This highlights the significance of C.1 and C.2 in the wholesome representation of the data. To give more weight to the importance of C.1 and C.2, the scree plot showed a strong inflection point at C.2. The importance of C.1 and C.2 in the description of the variables being investigated was highlighted by other graphical representations. The loading plot, as well as the biplot, assisted in breaking down the individual contribution of the key components to the variables. The interpretation of the loading and biplot figures enhanced how each principal component captures a distinct dimension of emission behavior. C.1 primarily captured the features related to CEs, TP, and EPOGS, thereby shedding light on their interrelationships. In contrast, C.2 predominantly explained the variance in the IVG variable. Importantly, our analysis demonstrated that both C.1 and C.2 collectively accounted for the patterns observed in EPCS and PGR. The study revealed that PGR and energy consumption are the primary factors influencing CO_2_ emissions, as indicated by their strong positive loadings on the first principal component. This illustrates the population‐driven and demand‐side dimension of emissions, reflecting the combined effect of demographic expansion and increased energy use.

The second principal component highlights the influence of industrial activities and the energy mix, indicating that industrial structure and energy composition are key contributors to emissions variability. This dual role highlights the significance of retaining C.1 and C.2 to achieve optimal data compression or dimensionality reduction while preserving the maximum variance inherent in the study variables. Additionally, the study advanced knowledge of the major factors influencing CO_2_ emissions and supports cleaner energy use, sustainable industrial growth, and effective climate policies, all of which are in line with SDG 7 (Affordable and Clean Energy), SDG 9 (Industry, Innovation, and Infrastructure), and SDG 13 (Climate Action). A comparative interpretation of the components suggests that both demographic and industrial factors jointly determine emission trends, underscoring the need for integrated mitigation strategies. The first component emphasizes the link between population, energy demand, and emissions, while the second reflects the industrial and technological dynamics within the energy sector. These findings provide deeper analytical insight into the underlying causes of emissions and offer practical guidance for designing policies that address both demand‐side efficiency and supply‐side sustainability.

### 4.1. Policy Implications

These findings offer information for mitigation strategies, though the data are country‐specific. In order to control overall emissions, policymakers should give priority to demand‐side energy efficiency, the growth of renewable energy sources, and population‐driven energy planning. This corroborates the first principal component, which is dominated by population, energy usage, and CO_2_ emissions. The necessity for sector‐specific decarbonization initiatives, such as the use of cleaner industrial fuels, the execution of energy audits, and a greater electrification of industrial processes, corroborates the second principal component, which reflects the industrial structure and energy mix.

Additionally, energy and climate policy are informed by the PCA findings. In relation to SDGs 7 and 13, the first component (population, energy use, and CO_2_ emissions) emphasizes the need for demand‐side efficiency, the expansion of renewable energy, and population‐driven planning. SDGs 9 and 13 are supported by the second component (industrial structure, energy mix), which emphasizes sector‐specific decarbonization, greener fuels, energy audits, and industrial electrification. These results convert statistical insights into practical methods for managing energy sustainably and mitigating climate change.

## Author Contributions

M.Q., M.I., M.D., I.H., K.T., F.K.B., R.K.A., and K.A.A. conceived the idea, planned the study, suggested the statistical methodology, drafted the manuscript, and reviewed the manuscript.

## Funding

No funding was secured for this study.

## Disclosure

All authors read and approved the final manuscript.

## Conflicts of Interest

The authors declare no conflicts of interest.

## Data Availability

The data for this study are from the World Bank’s data indicators available at https://databank.worldbank.org/country/PAK/556d8fa6/Popular_countries#selectedDimension_WDI_Ctry.
